# Editorial: Multimodality Monitoring or Evaluation of Neuro-Function in Modern NICU

**DOI:** 10.3389/fneur.2019.01423

**Published:** 2020-02-04

**Authors:** Liping Liu, Wengui Yu

**Affiliations:** ^1^Department of Neurology and Stroke Center, Beijing Tiantan Hospital, Capital Medical University, China National Clinical Research Center for Neurological Diseases, Beijing, China; ^2^Department of Neurology, University of California, Irvine, Irvine, CA, United States

**Keywords:** acute ischemic stroke, anoxic brain injury, encephalitis, multi-modality monitoring, outcome, prognostication, status epilepticus

There have been significant advances in the management of acute ischemic stroke (AIS), autoimmune encephalitis, and anoxic brain injury in recent years. This Research Topic, Multimodality Monitoring and Evaluation of Neuro-Function in Modern Neurological Intensive Care Unit (NICU), presents eight original research articles and two mini reviews to highlight the applicability of novel multimodality monitoring and clinical evaluation in the management and prognostication of severe neurological disorders.

## Non-invasive Monitoring for Acute Ischemic Stroke

Non-invasive electroencephalography (EEG) can capture sensory cortical neurons' entrainment to rhythmic sound stimuli known as the auditory steady-state response. The 40-Hz steady-state response (SSR) reflects early sensory processing and has the potential to differentiate disease severity. Wang et al. investigated the predictive value of 40-Hz SSR in patients with large hemispheric infarction. Bilateral abnormal 40-Hz SSRs were found to have a high specificity and positive predictive value for 30-day mortality and 90-day poor prognosis.

Neurovascular coupling is a dynamic physiological process that adjusts cerebral blood flow (CBF) to match neuronal activity needs. Liu et al. used continuous EEG and CBF velocity monitoring to investigate neurovascular coupling in patients with stroke from large vessel occlusion (LVO). A phase-amplitude cross-frequency coupling (CFC) algorithm was applied to assess how CBF velocities interact with the EEG amplitude. The degree of hemispherical asymmetry of CFC was found to correlate with the degree of arterial stenosis.

Huang et al. investigated the correlation between hyperchloremia and outcome in patients with severe stroke. Out of 405 study patients, 35 (8.6%) and 69 (17.0%) were found to have hyperchloremia at NICU admission and within 72 h, respectively. New-onset hyperchloremia and every 5 mmol/L increment in Cl^−^ were associated with increased 30-day mortality and poor 6-month outcome. However, hyperchloremia was not an independent predictor of poor outcome in multivariate models.

Taken together, EEG has great potential for evaluating LVO and outcomes from large hemispheric infarction. In contrast, hyperchloremia may be iatrogenic from the use of hypertonic saline in the NICU and is not an independent predictor of poor outcome.

## Defining Blood Pressure Goals During and After Recanalization Therapy

Extreme blood pressure (BP) and hemodynamic instability in stroke patients are associated with worse outcome. Vitt et al. provided an excellent overview of stroke pathophysiology and current BP management. Ideal BP target after recanalization therapy depends on the degree of reperfusion and extent of infarction. Following complete recanalization, lower BP target [i.e., systolic BP (SBP) < 140 mmHg] is reasonable to prevent reperfusion injury. However, randomized clinical trials are warranted to define optimal BP goals during and after recanalization therapy.

## Continuous Vital Sign Analysis to Predict Neurological Decline After Traumatic Brain Injury

In patients with traumatic brain injury (TBI), one of the major goals is to identify and prevent secondary neurological decline (ND). Melinosky et al. analyzed beat-to-beat variation of electrocardiogram (ECG) and photoplethysmogram (PPG) as well as waveform features during the first 15–60 min to identify physiologic parameters associated with future ND. Among 191 patients, 33 (17%) developed ND. Both ECG and PPG analyses during the first 15 min predicted ND better than did clinical characteristics. Predictive probability for ND by a PPG analysis at 15 min (*p* = 0.03) was independently associated with inpatient mortality. Early vital sign variation appears to be a promising biomarker of outcome prognostication in TBI.

## Targeted Temperature Management and Multimodality Monitoring

Nguyen et al. provided an excellent review of targeted temperature management (TTM) and multimodality monitoring in modern NICU. TTM is neuroprotective for patients with anoxic brain injury. Current guidelines recommend the use of TTM for comatose survivors after out-of-hospital cardiac arrest from a shockable rhythm. Neurointensivists are central in the patient evaluation, management, and prognostication. Established prognostic tools include clinical exam, somatosensory evoked potential (SSEP), EEG, and MRI. Currently, functional MRI and invasive monitoring are not validated in prognostication, and further studies on biomarkers of poor outcomes are warranted.

## Regional Cerebral Oximetry for Acute Brain Injury

Regional cerebral oxygen saturation (rScO_2_) measured by near-infrared spectroscopy (NIRS) can be used to monitor brain oxygenation and acute brain injury (ABI) in extracorporeal membrane oxygenation (ECMO). Khan et al. evaluated the association between rScO_2_ and ABI in ECMO patients. Among 18 study patients, 11 (61%) experienced rScO2 desaturations and 6 (33%) exhibited ABI. All ABI patients experienced rScO_2_ desaturation as compared with 42% patients without ABI (*p* = 0.04). The presence and burden of cerebral desaturations noted on NIRS cerebral oximetry are associated with secondary ABI in ECMO patients. Monitoring of rScO_2_ may be applicable in ECMO and TBI patients.

## Otoacoustic Emissions as Outcome Markers in Anoxic Brain Injury

Kondziella et al. assessed the usefulness of otoacoustic emissions as outcome markers for comatose patients after cardiac arrest. Distortion-product otoacoustic emissions (DPOAEs) and transient evoked otoacoustic emissions (TEOAEs) were measured in cardiac arrest patients and 10 patients with myocardial infarction as controls. Compared with controls, cardiac arrest patients had significantly less preserved DPOAE [9.2 vs. 40.8%; odds ratio (OR) 0.15 (CI 0.07–0.30); *p* < 0.0001]. TEOAEs were not statistically different between the two groups. Despite convenience, otoacoustic emissions were unreliable prognostic markers in cardiac arrest survivors.

## Predicting Outcome for Acute Encephalitis

Status epilepticus (SE) is the most serious complication of acute encephalitis. Early progression to super-refractory SE (SRSE) is associated with poor outcome. Yuan F et al. reported a retrospective study of 94 patients with SE from autoimmune encephalitis. There were 23.4% SRSE and 76.6% non-SRSE. Cortical or hippocampal abnormality on neuroimaging (*p* = 0.002, OR 20.55, 95% CI 3.16–133.46) and Encephalitis-Non-convulsive Status Epilepticus-Diazepam Resistance-Image Abnormalities-Tracheal Intubation (END-IT) score (*p* < 0.001, OR 4.07, 95% CI 1.91–8.67) were found to be independent predictors of progression to SRSE. Recurrent clinical or EEG seizures, tracheal intubation, and emergency resuscitation predict poor functional outcome.

Jiang et al. reported the use of quantitative EEG (qEEG) to predict the outcome of anti-*N*-methyl d-aspartate receptor (anti-NMDAR) encephalitis. Twenty-six patients were divided into critically ill (*n* = 14) and non-critically ill (*n* = 12) group on the basis of ICU admission. All patients underwent 2-h 10-channel qEEG recordings at the acute stage. No difference in qEEG parameters was observed between the two groups. A logistic regression analysis revealed that a narrower parietal amplitude-integrated EEG bandwidth was associated with favorable long-term outcomes (OR 37.9; *p* = 0.044).

These studies suggest that neuroimaging and qEEG may be used together to predict outcome of acute encephalitis.

In summary, multimodality monitoring has been rigorously investigated in patients with TBI and subarachnoid hemorrhage in the last two decades. Although invasive multimodality monitoring may provide evidence of pathophysiological changes in a local area near the implanted probe, it is associated with risk of periprocedural complication and low sensitivity for the detection of changes in other brain regions. The articles in this Research Topic collection have presented new insight on non-invasive multimodality monitoring and vital sign variability analysis in prognostication of patients with large hemispheric infarction, acute encephalitis, and acute brain injury. The collection has also included judicious appraisal of targeted temperature management after cardiac arrest and blood pressure goals after endovascular thrombectomy. Currently, it remains challenging to predict secondary brain injury and outcome. This Research Topic article collection will stimulate future research on the development of integrated and reliable non-invasive monitoring and clinical evaluation tools in modern NICU.

## Author Contributions

LL and WY contributed to the drafting of this Editorial for the Research Topic that they edited. WY made critical revision and finalized the editorial.

### Conflict of Interest

The authors declare that the research was conducted in the absence of any commercial or financial relationships that could be construed as a potential conflict of interest.

